# Non-redundant activity of GSK-3α and GSK-3β in T cell-mediated tumor rejection

**DOI:** 10.1016/j.isci.2021.102555

**Published:** 2021-05-19

**Authors:** Lynette Steele, Aarren J. Mannion, Gary Shaw, Kenneth A. Maclennan, Graham P. Cook, Christopher E. Rudd, Alison Taylor

**Affiliations:** 1 Leeds Institute of Medical Research, University of Leeds, School of Medicine, Wellcome Trust Brenner Building, St James's University Hospital, Leeds LS9 7TF, UK; 2 Department of Oncology-Pathology, Karolinska Institute, Stockholm, Sweden; 3 Division of Immunology-Oncology Research Center, Maisonneuve-Rosemont Hospital, Montreal, Quebec H1T 2M4, Canada; 4 Département de Medicine, Université de Montréal, Montreal, Quebec H3C 3J7, Canada; 5 Division of Experimental Medicine, Department of Medicine, McGill University Health Center, McGill University, Montreal, Quebec H4A 3J1, Canada

**Keywords:** cancer, cell biology, functional aspects of cell biology, immunology

## Abstract

Glycogen synthase kinase-3 (GSK-3) is a positive regulator of PD-1 expression in CD8+ T cells and GSK-3 inhibition enhances T cell function and is effective in the control of tumor growth. GSK-3 has two co-expressed isoforms, GSK-3α and GSK-3β. Using conditional gene targeting, we demonstrate that both isoforms contribute to T cell function to different degrees. *Gsk3b*−/− mice suppressed tumor growth to the same degree as *Gsk3a/b*−/− mice, whereas *Gsk3a−/−* mice behaved similarly to wild-type, revealing an important role for GSK-3β in regulating T cell-mediated anti-tumor immunity. The individual GSK-3α and β isoforms have differential effects on PD-1, IFNγ, and granzyme B expression and operate in synergy to control PD-1 expression and the infiltration of tumors with CD4 and CD8 T cells. Our data reveal a complex interplay of the GSK-3 isoforms in the control of tumor immunity and highlight non-redundant activity of GSK-3 isoforms in T cells, with implications for immunotherapy.

## Highlights

• Conditional knockouts reveal a non-redundant role for GSK-3α and GSK-3β in T cells.
• GSK-3α and GSK-3β act together to enhance PD-1 expression.
• GSK-3β depletion alone enhances T cell mediated tumor rejection.
• GSK-3β plays an important role in regulating T cell-mediated anti-tumor immunity.

## Graphical abstract

**Figure:**
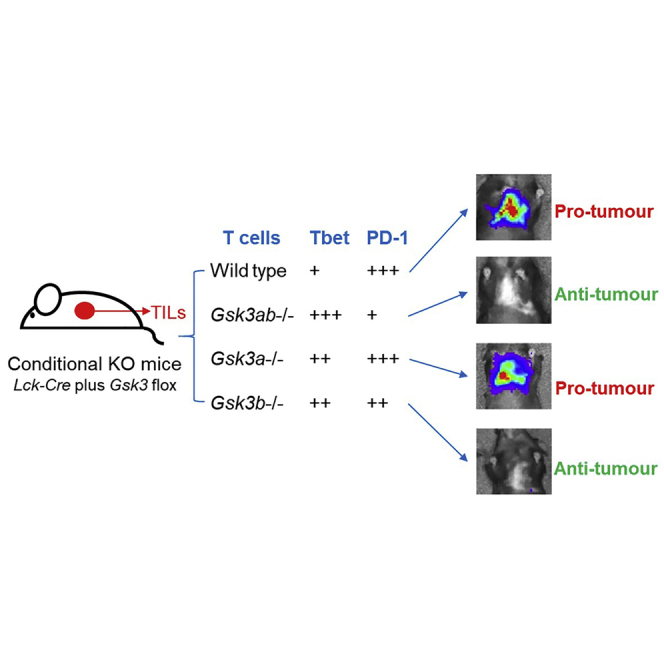

## Introduction

Glycogen synthase kinase-3 (GSK-3) was first discovered in 1980 as a regulatory kinase which phosphorylates and inhibits glycogen synthase (Embi et al., 1980) and has since been implicated in several disease states and key cellular processes, including Wnt, insulin, and Hedgehog signaling (Matthews and Cantrell, 2009). Two isoforms of GSK-3 have been reported in mammals; a 51 kDa GSK-3α and a 47 kDa GSK-3β isoform, encoded by the unlinked *Gsk3a* and *Gsk3b* genes, respectively. These two isoforms exhibit 98% homology in their kinase domains but only 36% identity in the last 76 C-terminal amino acid residues. Both isoforms are highly expressed in many tissues (Woodgett, 1990) and have been implicated in processes ranging from glycogen metabolism to gene transcription, apoptosis and microtubule stability (Eldar-Finkelman and Martinez, 2011; Frame and Cohen, 2001). GSK-3β is thought to be of prime importance in diabetes (McManus et al., 2005), Alzheimer disease (Phiel et al., 2003), and inflammation (Martin et al., 2005).

An unusual aspect of GSK-3 is that it is constitutively active in resting cells (Embi et al., 1980; Woodgett, 1990) and its inactivation occurs through phosphorylation of specific serine residues (Ser9 in GSK-3β, Ser21 in GSK-3α) (Hughes et al., 1993; Rayasam et al., 2009). This phosphorylation allows the phosphoserine tail of GSK-3 to bind and block its own active site (Doble and Woodgett, 2003; Rayasam et al., 2009). In contrast to this, tyrosine phosphorylation of GSK-3 (Tyr216 in GSK-3β, Tyr279 in GSK-3α) enhances its ability to bind and phosphorylate substrates (Frame and Cohen, 2001; Hughes et al., 1993). Furthermore GSK-3 has a preference for substrates which have already been phosphorylated by a “priming kinase” (Picton et al., 1982). For example, glycogen synthase is primed by casein kinase 2 (CK2) prior to its subsequent phosphorylation and inactivation by GSK-3 (Picton et al., 1982).

GSK-3 can phosphorylate more than one hundred substrates (Sutherland, 2011) and plays a key role in T cell activation (Ohteki et al., 2000; Rudd et al., 2020; Taylor et al., 2016; Taylor and Rudd, 2020). Active GSK-3 blocks T cell activation and cytokine production (Ohteki et al., 2000), and we previously showed that the inhibition of GSK-3 downregulate PD-1 and LAG-3 gene expression (Taylor et al., 2016). Other substrates include transcription factors such as cyclic AMP response element binding protein, the nuclear factor of activated T cells (NFATs), β-catenin, c-Jun, and NF-κB (Cohen and Frame, 2001; Eldar-Finkelman and Martinez, 2011; Grimes and Jope, 2001). In the case of NFAT, GSK-3 inactivates the pathway by phosphorylating NFAT and facilitating its exit from the nucleus in T cells (Beals et al., 1997; Neal and Clipstone, 2001). Active GSK-3 inhibits T cell proliferation (Ohteki et al., 2000), whereas T cell receptor (TCR) and CD28 ligation induces GSK-3 phospho-inactivation (Appleman et al., 2002; Ohteki et al., 2000; Wood et al., 2006) dependent on phosphatidylinositol 3-kinase (PI3-K) (Taylor and Rudd, 2017).

As a regulator of PD-1 and LAG3 expression, we previously showed that small molecule inhibitors (SMIs) and siRNA down-regulation of GSK-3 are effective in promoting viral clearance (Taylor et al., 2016) and suppressing tumor growth (Taylor et al., 2018). Mechanistically, this was found to operate by enhancing Tbet (*Tbx21*) transcription which, in turn, inhibits *Pdcd1* gene expression by repressing the *Pdcd1* promoter (Hui et al., 2017; Rudd et al., 2020; Taylor et al., 2016; Taylor & Rudd, 2017, 2019). Tbet also regulates an array of other genes, including cytokines such as interleukin-2 and effector proteins such as granzyme B which are needed for optimal CD8 cytolytic function (Lazarevic and Glimcher, 2011; Sullivan et al., 2003). An unanswered question concerns the relative roles of the two isoforms of GSK-3 in the modulation of PD-1 and protective immunity against cancer. Here, we show the alpha and beta isoforms differentially regulate PD-1, IFNγ and Granzyme B expression, whilst deletion of both isoforms synergizes to reduce PD-1 expression and promote the T cell infiltration into tumors.

## Results

### Conditional knockout of either or both isoform(s) of GSK-3 does not affect the total number of splenic T cells

**Figure 1:**
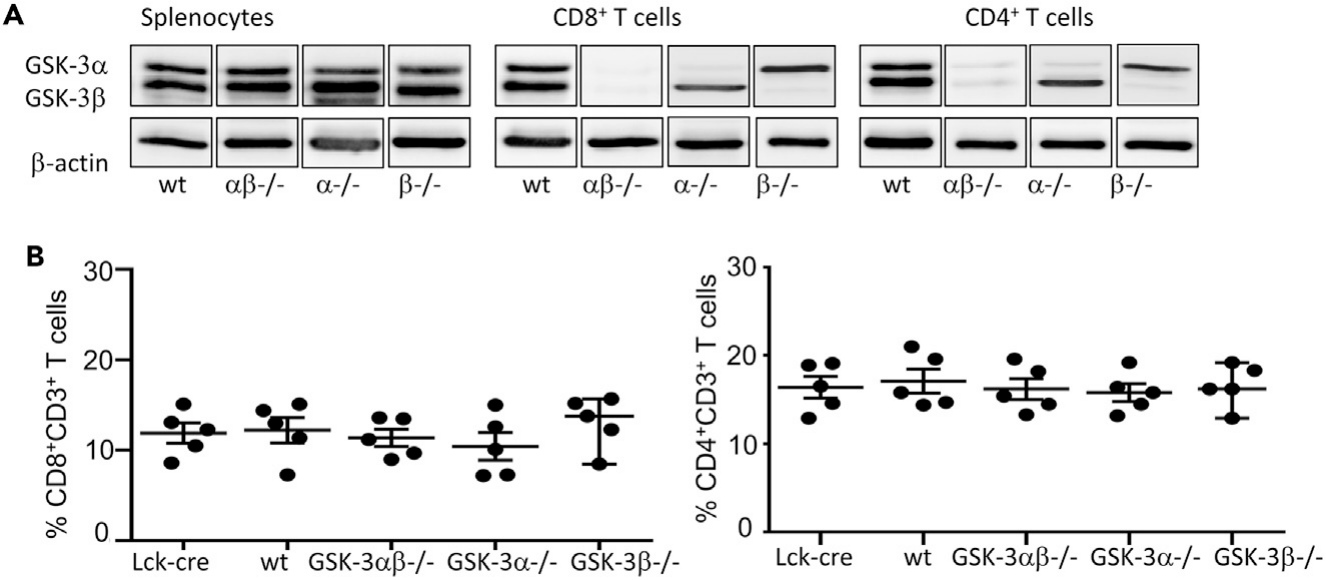
Conditional knockout of GSK-3 alpha or GSK-3 beta either alone or together does not affect the levels of CD4 or CD8+ T cells (A) Western blot depicting expression of GSK-3 isoforms; alpha or beta in spleen cells (left panel), purified CD8+ T cells (middle panel) and purified CD4+ T cells (Right panel) taken from the spleens of GSK-3 conditional knockout mice and wt littermates. (B) Percentage of CD8+ (left) or CD4+ (right) CD3 T cells in the spleens of conditional knockout mice, wt littermates and Lck-cre control mice, as determined by flow cytometry. Data are represented as mean ± SEM.

Our previous studies have demonstrated a clear role for GSK-3 in the regulation of tumor growth, with the inhibition of GSK-3 through SMIs potentiating T cell reactivity leading to diminished growth (Rudd et al., 2020; Taylor et al., 2018). However, it is unclear if the different isoforms of GSK-3 work in a similar manner, if both isoforms are required for the function of GSK-3, or if the action of one is dominant over the other, particularly in the context of cancer. Several SMIs are available for GSK-3 and cited *k*_*i*_ values suggest that it is possible for SMIs to preferentially target one isoform over the other; although in practice, particularly *in vivo*, evidence suggests that this is not the case and that inhibitors tend to act against both isoforms, albeit at varying levels (Lo Monte et al., 2012; Martinez and Perez, 2008). To unequivocally answer this question, we have used *Gsk3α*-flox/flox-*β*−flox/flox mice crossed with an Lck-Cre line (Jackson labs) which uses the distal Lck promoter to delete both *Gsk3a* and *Gsk3b* in post-selection CD4+CD8+ DP cells without affecting the T cell repertoire (Chiang and Hodes, 2016) to generate *Gsk3ab* cKO mice. These mice were then used further to generate individual α and β isoform-specific cKO mice; *Gsk3a*-flox/flox-Lck-Cre+ (*Gsk3a* cKO) and *Gsk3b*-flox/flox-Lck-Cre+ (*Gsk3b* cKO), respectively, resulting in mice with T cells devoid of either GSK-3α or GSK-3β (Figure 1). This was apparent in both CD4 and CD8 T cell populations as shown in Figure 1A. Importantly, the loss of GSK-3 expression in T cells had no effect on the total number of CD8+ and CD4+ splenic T cells when compared to control mice (Figure 1B).

### T cell depletion of *Gsk3b*, alone or in combination with *Gsk3a* suppresses growth of two distinct tumor types

**Figure 2:**
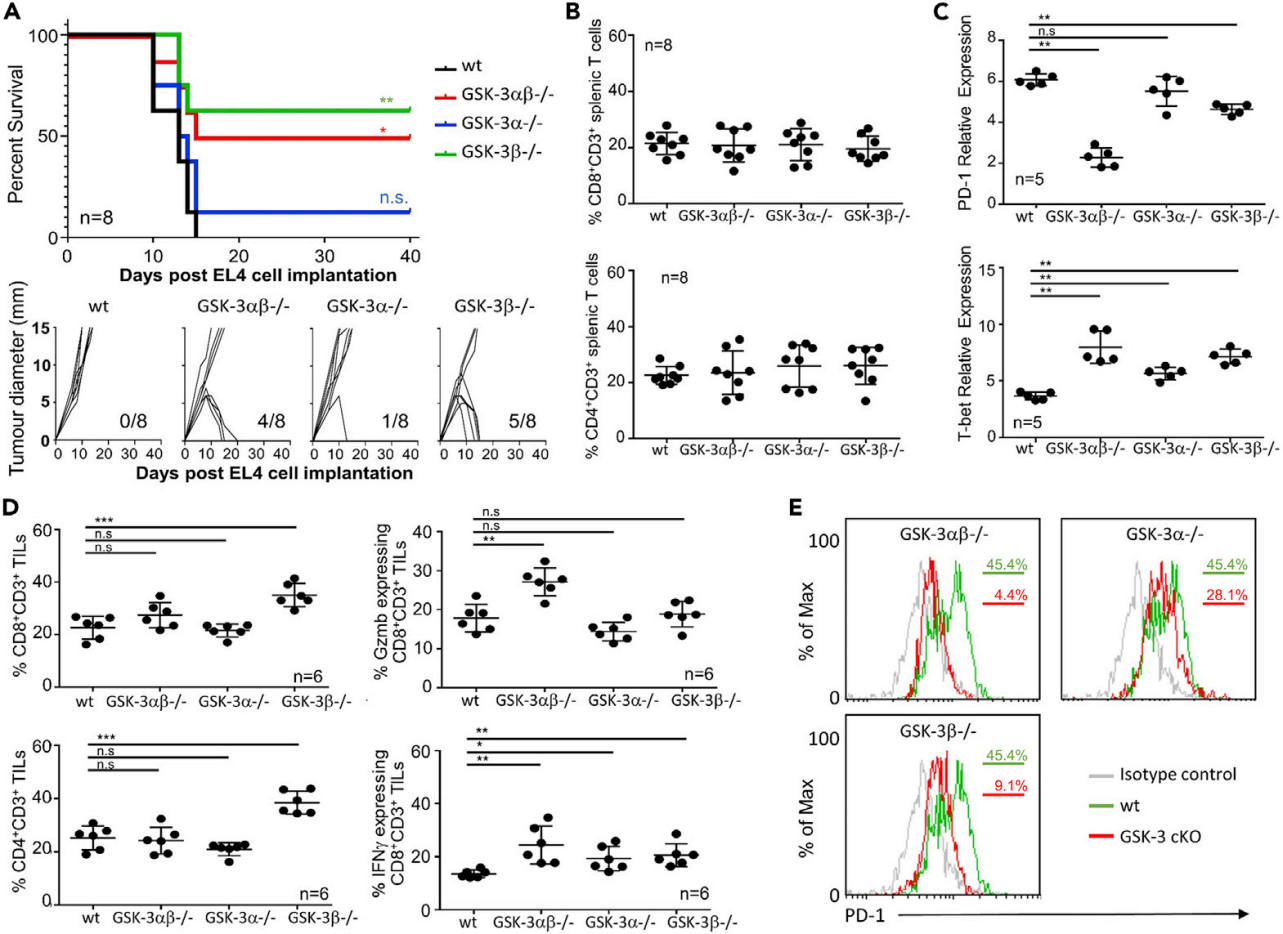
Depletion of either the GSK-3 beta isoform alone or in combination with GSK-3-alpha in T cells can protect against EL4 tumor progression (A) Survival plot (upper panel) of conditional knockout mice as indicated following EL4 tumor cell implantation. Growth curves of EL4 tumors implanted into GSK-3 cKO mice (lower panel). Numbers in bottom right corner indicate the number of tumor-free mice after 40 days. n = 8. (B) Percentage of CD8 (upper panel) and CD4 (lower panel) CD3+ T cells in the spleen as determined by flow cytometry. (C) Quantitative real-time PCR of splenic T cells taken from animals at day 10. (D) Percentage of CD8+ (upper left panel), CD4+ (lower left panel), granzyme B (Gzmb) expressing CD8+ (upper right panel) and IFNγ expressing CD8+ tumor-infiltrating cells (TILs) (taken from animals at day 10) as determined by flow cytometry. (E) Flow cytometric profiles for T cells isolated from tumor-infiltrating cells (TILs) (data taken from animals at day 10 and representative of 5 samples). Data represent three combined independent experiments of n ≥ 5 mice per group. Data are represented as mean ± SEM. Groups compared using unpaired t test. ∗∗∗p < 0.0001; ∗∗p < 0.001; ∗p < 0.01; ns, no significant difference relative to controls. Log rank tests performed for Kaplan-Meier survival data.

Previously, we showed that using GSK-3 SMIs in *in vivo* mouse models of cancer led to suppression of tumor growth (Taylor et al., 2018). These studies focused on the CD8+ T cell population as the effector cells. However, SMIs act across all tissues and although CD8+ T cells were investigated *ex vivo* there is still the possibility that other cells might play a role within these models. We have now utilized the newly generated cKO mice to test if GSK-3 deletion in T cells alone is sufficient to suppress tumor growth and whether deletion of both isoforms is required. We first determined the tumor growth rate and survival kinetics in the three different strains of cKO mice bearing EL4 tumors in the flank (Figure 2A) The mean survival time of 13 days for wt littermate mice was similar to that seen in *Gsk3a* cKO mice, with the exception of 1 mouse surviving of 8. In contrast, depletion of *Gsk3b* alone or in combination with *Gsk3a* gave a clear increase in the overall survival rate, with 50% of *Gsk3ab* cKO mice and 60% of *Gsk3b* cKO still surviving at day 40. This survival was concurrent with diminished tumor growth in these animals and the eventual eradication of tumor which was apparent within 15–20 days post-tumor challenge (lower panel Figure 2A). In a repeat study, EL4 tumor bearing mice were sacrificed at day 10 (when tumors were present in all mice) to analyze splenic and tumor-infiltrating cells. In agreement with the results from tumor-free animals (Figure 1B), no difference was seen in the frequency of splenic CD4+ or CD8+ T cells between the different cKO mice bearing EL4 tumors (Figure 2B). Previous studies using GSK-3 SMIs have shown that control of tumor growth is associated with a decrease in PD-1 expression, correlated with an increase in the expression of the transcription factor T-bet (Taylor et al., 2016). Here, using quantitative real-time polymerase chain reaction (qRT-PCR) of splenic CD8+ T cells, we found *Pdcd1* gene expression to be significantly lower in the *Gsk3ab* cKO compared to wt littermates, and this was associated with increased *Tbx21* expression (Figure 2C). A decrease in *Pdcd1* expression was also seen in the *Gsk3b* cKO, whilst statistically significant, the reduction in expression of *Pdcd1* in *Gsk3b* cKO T cells was not as great as that seen in the double cKO. There was no effect on *Pdcd1* expression in *Gsk3a* cKO cells but all three cKO strains showed increased *Tbx21* gene expression (Figure 2C).

Analysis of the tumors revealed no difference in the infiltration of CD4+ or CD8+ T cells in the double cKO mice when compared to wt littermates, in agreement with results obtained using GSK-3 SMIs (Taylor et al., 2018). However, deletion of *Gsk3b* alone resulted in significantly increased infiltration of both CD4+ and CD8+ T cells (Figure 2D). Tumor-infiltrating CD8+ T cells from the double cKO mice had significantly higher levels of granzyme B expression and increased numbers of IFNγ expressing cells. Although the latter was true for all three strains of GSK-3 cKO, the levels of granzyme B were only increased when both GSK-3 isoforms were depleted (Figure 2D). These findings indicate that the regulatory effects of GSK-3 on these events are more nuanced that previously appreciated. It appears the required events that determine tumor infiltration are different from those needed for the differentiation of T cells leading to Granzyme B expression. Unlike these differences, cell surface PD-1 expression was measured on tumor-infiltrating lymphocytes (TILs) and showed a clear reduction in the double and *Gsk3b* cKO mouse cells (Figure 2E) and a lesser reduction in TILs from the *Gsk3a* cKO mice, consistent with the *Pdcd1* gene expression data from the spleen (Figure 2C).

**Figure 3:**
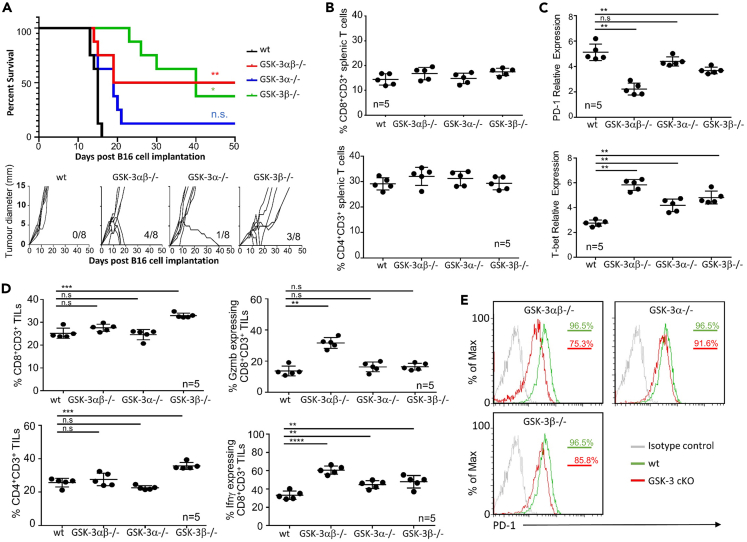
Depletion of either the GSK-3 beta isoform alone or in combination with GSK-3-alpha in T cells can protect against B16 flank tumor progression (A) Survival plot (upper panel) of conditional knockout mice as indicated following B16 tumor cell implantation. Growth curves of EL4 tumors implanted into GSK-3 cKO mice (lower panel). Numbers in bottom right corner indicate the number of tumor-free mice after 50 days. n = 8. (B) Percentage of CD8 (upper panel) and CD4 (lower panel) CD3+ T cells in the spleen as determined by flow cytometry. (C) Quantitative real-time PCR of splenic T cells taken from animals at day 15. (D) Percentage of CD8+ (upper left panel), CD4+ (lower left panel), granzyme B (Gzmb) expressing CD8+ (upper right panel) and IFNγ expressing CD8+ tumor-infiltrating cells (TILs) (taken from animals at day 15) as determined by flow cytometry. E) Flow cytometric profiles for PD-1 expressing T cells isolated from tumor-infiltrating cells (TILs) taken from animals at day 15 (data representative of 5 samples). Data represent three combined independent experiments of n ≥ 5 mice per group. Data are represented as mean ± SEM. Groups compared using unpaired t test. ∗∗∗p < 0.0001; ∗∗p < 0.001; ∗p < 0.01; ns, no significant difference relative to controls. Log rank tests performed for Kaplan-Meier survival data.

We next sought to confirm the results obtained from cKO mice bearing EL4 tumors with those bearing a second tumor type, B16 melanoma, and to determine whether deletion of one GSK-3 isoform could lead to suppression of both flank tumors and pulmonary metastasis. B16 tumor cells tagged with luciferase were injected intradermally into the three different strains of cKO mice and control animals and the tumor growth rate and survival kinetics assessed. The wt littermate mice and *Gsk3a* cKO mice bearing B16 flank tumors demonstrated a mean survival of 15 and 18 days, respectively, with the exception of 1 surviving mouse of 8 in the *Gsk3a* cKO (Figure 3A). In contrast, the double GSK-3 cKO demonstrated a 50% survival rate at day 50. This was slightly lower in the *Gsk3b* cKO, with 40% of mice surviving at day 50; here increased survival was associated with diminished tumor growth and the eventual eradication of tumor was apparent within 15–20 days post-tumor challenge (lower panel Figure 3A). It should be noted that there was an overall prolonged survival of the *Gsk3b* cKO mice of 5–10 days compared to all other strains, with tumor growth being slower in these mice (Figure 3A).

Splenic and tumor-infiltrating cells were analyzed from B16 flank tumor bearing mice which were sacrificed at day 12 (a time point at which tumors were present in the majority of animals to enable TIL isolation). As expected, no difference was seen in the number of splenic CD4^+^ or CD8^+^ T cells from the different cKO mice compared to wt littermates (Figure 3B). qRT-PCR of splenic CD8^+^ T cells demonstrated *Pdcd1* transcription to be significantly lower in the double GSK-3 cKO compared to wt littermates, and this was associated with increased *Tbx21* gene expression (Figure 3C). Furthermore, as seen in the EL4 lymphoma model, a significant decrease in *Pdcd1* gene expression was also seen in the *Gsk3b* cKO but not in *Gsk3a* cKO cells (Figure 3C). All three cKO strains showed increased *Tbx21* gene expression.

The *Gsk3b* cKO had a significantly higher number of infiltrating CD4+ and CD8+ T cells compared to the other cKOs and wt littermates (Figure 3D). Further analysis demonstrated tumor-infiltrating cells from GSK-3 double cKO mice to have higher levels of granzyme B expression compared to the single cKO mice. All three strains of GSK-3 cKO had increased levels of IFNγ expressing TILs compared to wt littermates. Finally, cell surface PD-1 expression on TILs showed a clear reduction in the double cKO mice cells (Figure 3E), some reduction in the *Gsk3b* cKO and little change in the *Gsk3a* cKO, in agreement with *Pdcd1* gene expression changes observed in spleen (Figure 3C).

**Figure 4:**
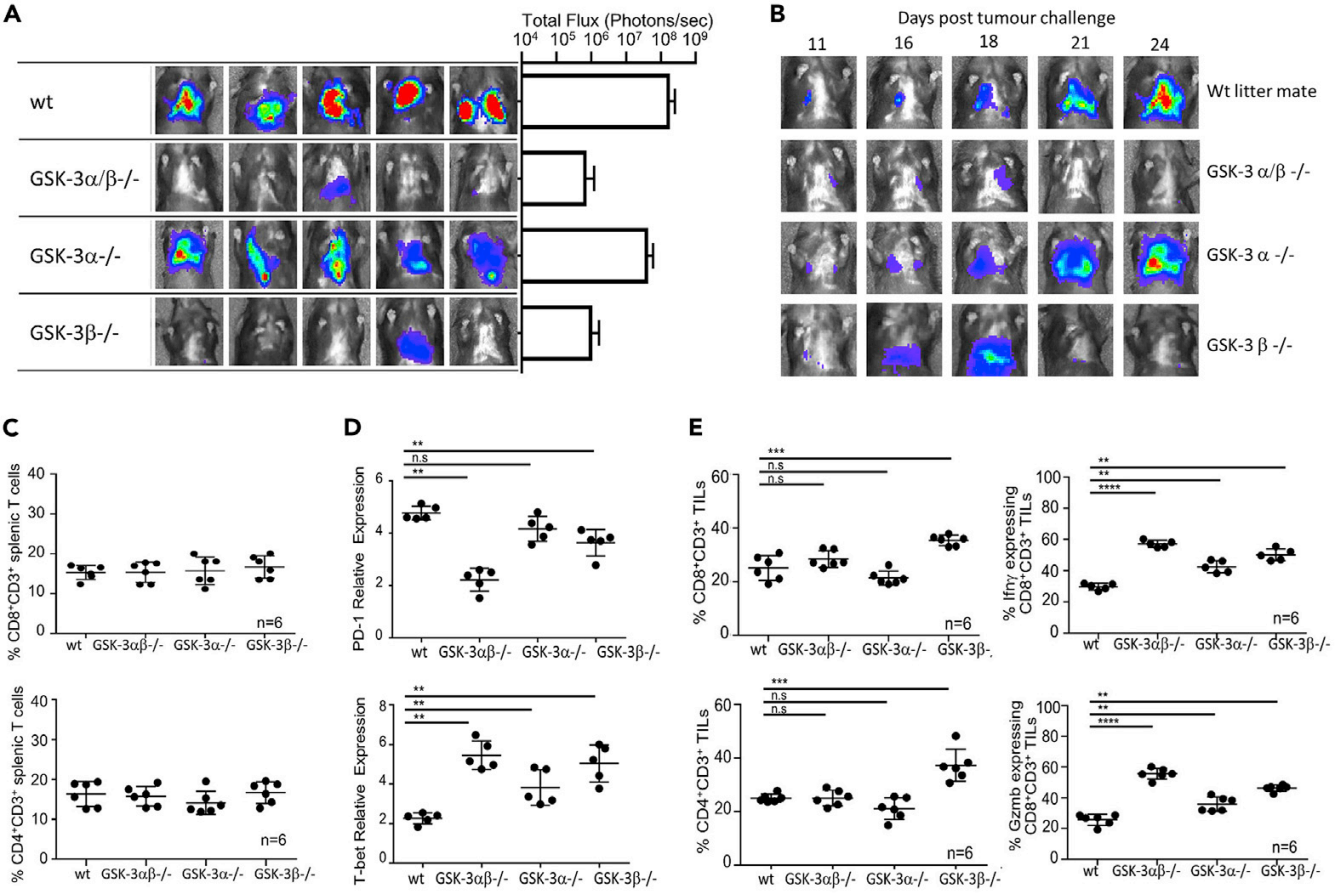
Depletion of either the GSK-3 beta isoform alone or in combination with GSK-3-alpha in T cells can protect against pulmonary metastasis of B16 melanoma (A) luminescent image (upper panel) showing B16 metastasis at day 24 with total flux (photons/second) values depicted in the histogram to the right. (B) Luminescent imaging of conditional knockout mice at time points indicated following B16 tumor cell implantation. (C) Percentage of CD8 (upper panel) and CD4 (lower panel) CD3+ T cells in the spleen as determined by flow cytometry. (D) Quantitative real-time PCR of splenic T cells taken from animals at day 18. (E) Percentage of CD8+ (upper left panel), CD4+ (lower left panel), granzyme B (Gzmb) expressing CD8+ (upper right panel) and IFNγ expressing CD8+ tumor-infiltrating cells (TILs) (taken from animals at day 18) as determined by flow cytometry. ∗∗∗p < 0.0001; ∗∗p < 0.001; ∗p < 0.01; ns, no significant difference relative to controls. Data represent three combined independent experiments of n ≥ 5 mice per group. Data are represented as mean ± SEM. Groups compared using unpaired t test. ∗∗∗p < 0.0001; ∗∗p < 0.001; ∗p < 0.01; ns, no significant difference relative to controls.

The data from both the EL4 (Figure 2) and B16 (Figure 3) flank tumors demonstrate non-redundant activity of the GSK-3α and GSK-3β isoforms, with genetic inactivation of GSK-3β resulting in enhanced rejection of both tumor types in the flank. Human cutaneous melanoma metastasizes to multiple sites, including the lungs and the B16 model can seed tumors in the lung following tail vein injection. We therefore analyzed the requirement for T cell GSK-3 isoforms in B16 pulmonary metastasis in our cKO mice. Luciferase tagged B16 tumor cells were injected intravenously into the different cKO and control mice. At day 24, mice were injected intraperitoneally with luciferin and scanned by IVIS Lumina imaging; data from five mice from each group are shown along with the total flux measurements from these groups (Figure 4A). As found in the EL4 and B16 flank models, rejection of B16 pulmonary tumors was enhanced by deletion of GSK-3β, revealed by the *Gsk3b* cKO and double cKO mice but not the *Gsk3a* cKO mice (Figure 4A). This was shown further by tracking tumor spread within the lung at various timepoints (Figure 4B). This method also aided in the tissue collection from mice, ensuring that spleen and tumor samples were taken when tumors were apparent in all the different strains (Day 18). As found in the flank models, no difference was seen in the number of splenic CD4^+^ or CD8^+^ T cells between the different cKO mice (Figure 4C). Splenic CD8^+^ T cells from the double cKO and *Gsk3b* cKO mice showed the expected reduction in *Pdcd1* gene expression and all three strains of cKO mice showed increased *Tbx21* expression (Figure 4D). Analysis of TILs revealed increased infiltration of CD4+ and CD8+ T cells in the *Gsk3b* cKO mice and all three strains of cKO mice exhibited an increased frequency of CD8+ cells expressing granzyme B or IFNγ compared to controls (Figure 4E). Collectively, these data demonstrate a key role for the GSK-3β isoform in controlling T cell mediated anti-tumor immunity.

### Tumor rejection in *Gsk3b* cKO is associated with higher numbers of tumor-infiltrating T cells

**Figure 5:**
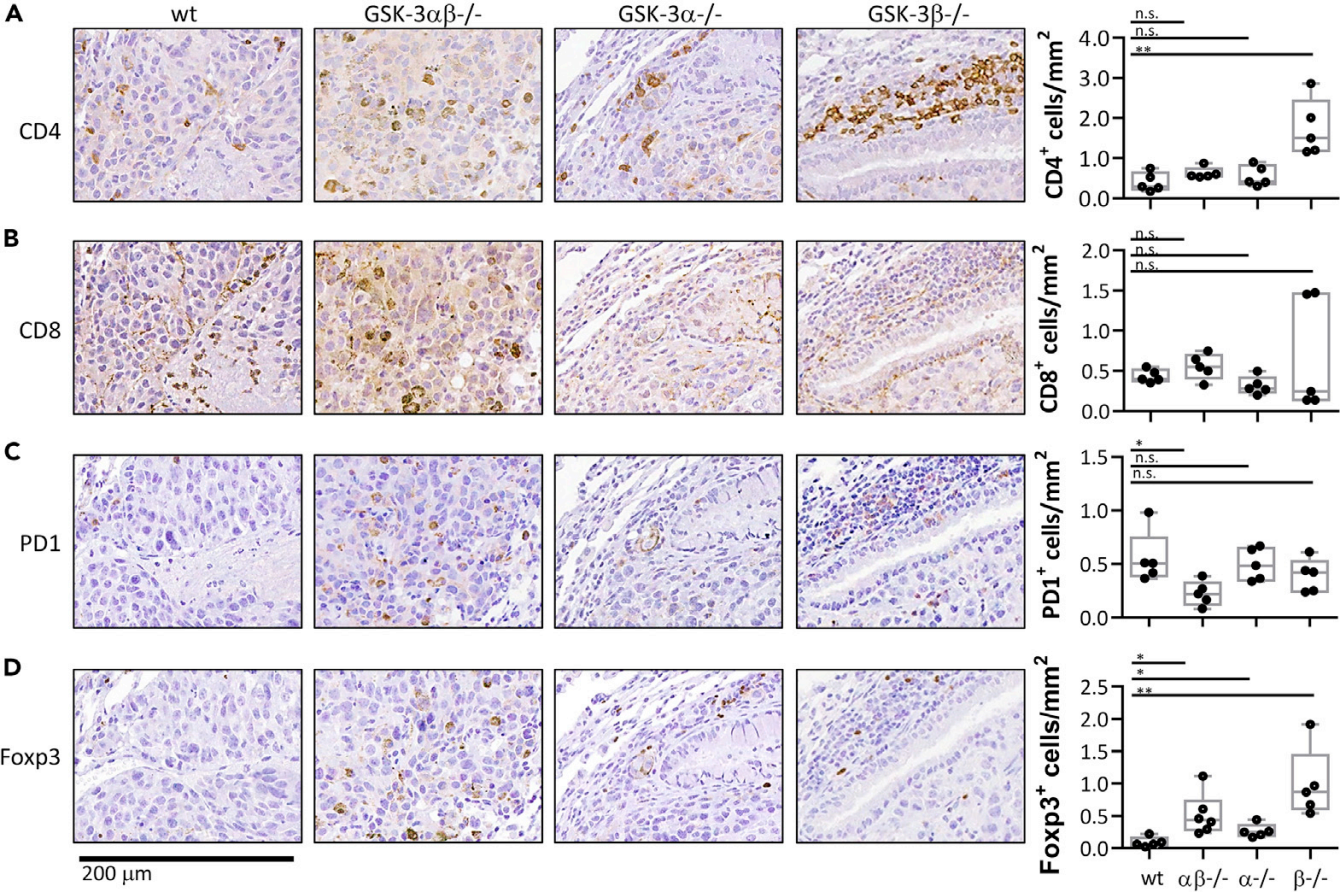
Tumor rejection in *Gsk-3b* cKO is associated with higher numbers of tumor-infiltrating T cells (A–D) Immunohistochemistry of lung tissue sections from B16 tumor bearing mice. Intra-tumoural areas are shown following staining for (A) CD4+, (B) CD8+, (C) PD1+ and (D) FOXP3+ expression. End panels depict number of positive cells quantified from 5 different areas using ImageScope Software. Data representative of two independent experiments. Groups compared using unpaired t test. ∗∗∗p < 0.0001; ∗∗p < 0.001; ∗p < 0.01; ns, no significant difference relative to controls.

Mice injected intravenously with B16 melanoma cells were culled 18 days later and lungs removed for immunohistochemistry (Figure 5). This timepoint was chosen to ensure that all mice were tumor bearing as confirmed prior to harvest by *in vivo* imaging. Staining showed CD4^+^ and CD8^+^ T cells to be scattered throughout the tumor region (Figures 5A and 5B, respectively) however, significantly increased numbers of CD4^+^ T cells were apparent in the *Gsk3b* cKO sections as determined using Imagescope software (Figure 5A, right panel). Further staining demonstrated all strains to contain PD-1 expressing cells throughout the tumor (Figure 5). A slight decrease in PD1^+^ cells was observed in the *Gsk3b* cKO, but this reduction was only significant when both isoforms were depleted in the double cKO. Interestingly, Foxp3 expression was significantly increased in all three cKOs compared to wt littermates.

**Figure 6:**
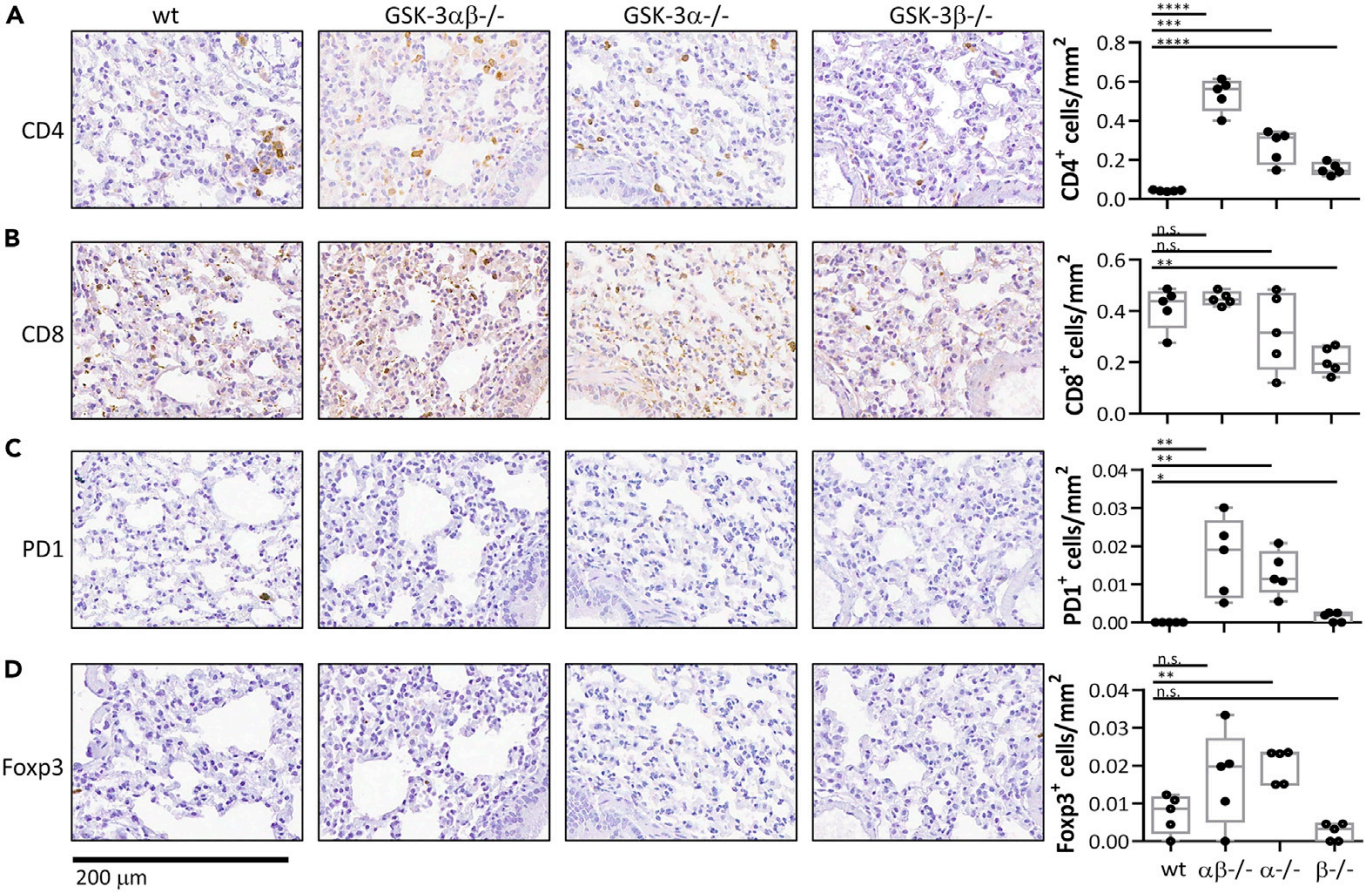
Reduced number of T cells in non-tumoural regions in b16 melanoma bearing *Gsk-3b* cKO (A–D) Immunohistochemistry of lung tissue sections from B16 tumor bearing mice. Non-tumor areas are shown following staining for (A) CD4+, (B) CD8+, (C) PD1+ and (D) FOXP3+ expression. End panels depict number of positive cells quantified from 5 different areas using ImageScope Software. Data are representative of two independent experiments. Groups compared using unpaired t test. ∗∗∗p < 0.0001; ∗∗p < 0.001; ∗p < 0.01; ns, no significant difference relative to controls.

T cell distributions within the peribronchial regions (areas surrounding the tumors rather than within the tumor itself) were also examined revealing accumulations of CD4^+^ T cells in the double cKO and *Gsk3a* cKO but not the *Gsk3b* cKO mice (Figure 6A). Furthermore, there was a significant reduction in CD8^+^ T cells in the *Gsk3b* cKO mice (Figure 6B). This reduction in peribronchial CD4+ and CD8+ T cells in the *Gsk3b* cKO mice was also reflected in lower numbers of Foxp3 and PD1 expressing cells (Figures 6C and 6D). Taken together, these data demonstrate a clear difference in T cell distribution in response to the presence of tumor growth with a higher level of tumor-infiltrating T cells being seen in the *Gsk3b* cKO mice.

Further to this, lung tissue was taken from a single *Gsk3b* cKO mouse which had completely rejected the B16 tumor by day 18 and IHC performed (Figure S1). There was a significant increase in the number of CD4^+^ T cells and Foxp3 expressing cells in the lungs of this mouse that rejected the tumor. This suggests that there is an increase in the cells migrating to the tumor site within *Gsk3b* cKO mice and that they efficiently infiltrate any tumors and contribute to controlling disease progression.

## Discussion

Despite our establishment of the importance of GSK-3 in the activation of T cell function needed for tumor rejection, the relative roles of the different GSK-3 isoforms in tumor rejection have been unclear. Our results demonstrate that GSK-3beta activity in T cells provides a more permissive environment for the tumor models tested here and that deletion of this isoform alone can enhance T cell mediated tumor rejection. However, the mechanism behind this remains to be fully elucidated. We have previously shown that inhibition of GSK-3 using SMIs leads to down regulation of checkpoint molecules PD-1 and LAG-3, whilst upregulating Tbet (Rudd et al., 2020; Taylor et al., 2016). These findings are supported here when genes encoding both GSK-3 isoforms are targeted in a cKO model. Furthermore, we show that the two isoforms contribute in different ways to the various T cell functions linked to tumor rejection. Overall, the GSK-3β isoform appeared to be dominant over the GSK-3α isoform, although both isoforms were needed for optimal tumor rejections.

The targeting of the GSK-3β isoform alone also provided a tumor suppressive effect, but the mechanism behind this may not be the same. There is some reduction in tumor growth, but this is not as striking as that seen when both isoforms are depleted. This suggests that deletion of the GSK-3α isoform is also needed for optimum suppression of tumor growth, although depletion of the GSK-3α isoform alone failed to show a phenotype. Whether its contribution was additive in promoting T cell rejection or it operated cooperatively is not clear. It is possible that the function of the GSK-3β is in some manner cross-regulated by the GSK-3α isoform but how both isoforms operate remains unclear. Similar to tumor rejection, depletion of *Gsk3a* alone, unlike *Gsk3b*, has no direct effect on PD-1 expression, whereas depletion of both together caused the effect to be magnified. One possibility is that the GSK-3β isoform is directly involved in the PD-1 pathway, whereas GSK-3α may act indirectly, affecting another protein which then acts on PD-1 expression. As, GSK-3α alone has no obvious effect on PD-1 expression, this would suggest that the protein it does act upon is also regulated by GSK-3β and therefore, the effects are only seen when both isoforms are switched on or off.

Another surprising finding was the suggestion that the two isoforms might differentially affect the CD4 and CD8 TILs in mice. CD4^+^ T cells were found to be at higher levels in tumors in *Gsk3b* cKO mice compared to the levels seen in *Gsk3a* cKO mice. More surprisingly, these levels were also higher than those seen in the double cKO mice. Although the increase in the numbers of infiltrating CD8+ T cells did not reach significance, it was apparent that some tumor regions had very high levels of CD8+ T cells, whilst others had very low levels. It may be speculated that had the samples been taken over a time course, an increase in T cell infiltration would have been more apparent and that perhaps this time point reflects a point at which CD8+ T cells were moving into the tumor site to attack tumor cells, or indeed they were already migrating away from these sites after fulfillment of their role. As seen in 40% of these cKO mice, a later time point may have resulted in no tumor being present and therefore it is possible that this is the recession following the peak of the immune response. Further analysis into the dynamics of T cell migration and infiltration are warranted to fully understand this aspect of GSK-3 regulation, as are a detailed analysis of the activation and maturation status of T cells in the different GSK-3 backgrounds; however, this is beyond the scope of the current manuscript. Regardless of these dynamics, the comparison of the infiltration patterns between these cKO mice at a specific time point suggests that the beta isoform plays a dominant role in controlling cellular movement and T cell migration. This finding is consistent with our previous work demonstrating the inhibition of GSK-3 (by SMIs) reduces motility and increase cell-to-cell contact, this coupled with enhanced CTL potential, led to tumor suppression (Taylor and Rudd, 2020).

The results showing higher tumor infiltration of T cells due to the beta isoform may be due to chemotactic effects enhancing migration of these T cells to the tumor site, or T cell retention at the tumor site, resulting in prolonged contact and efficient killing of the tumor cells. Oddly, this effect is not seen in the *Gsk3a* cKO mice, nor in the double cKO which suggests a possible opposing effect of the alpha isoform. The data outlines a possible complex interplay between the two isoforms, each playing their own part in different pathways, but yet together synergizing to reduce PD-1 expression, an effect which was optimally seen when both isoforms are depleted.

Previously our work has focused on CD8 T cells, more specifically cytotoxic T cells and their enhanced ability to kill tumor cells when GSK-3 is inactivated. However, these results clearly demonstrate that the GSK-3β isoform also impacts upon CD4+ T cells in the context of tumor immunity. Previous work has shown that GSK-3β is important for the differentiation of Th17 cells (Beurel et al., 2011), whilst GSK-3α is needed for Th1 cells (Beurel et al., 2013). Further investigation into the role of both isoforms in the different subsets is warranted to fully understand the mechanisms behind GSK-3 regulation in controlling the immune response. Such studies could potentially lead to new targeted therapeutic approaches.

### Limitations of the study

This study focused on determining the relative contributions of the two isoforms of GSK-3 on T cell-mediated tumor immunity. Our investigation extended to CD8+ T cells and the expression of PD-1 and Tbet by these cells as indicated from our previous work using GSK-3 inhibitors. Our results reveal both overlapping and non-redundant functions of the isoforms, but the detailed mechanisms by which the two isoforms regulate the T cell phenotype remain unclear at this time. A detailed understanding of the mechanisms by which GSK-3 regulates T cell activity requires analysis of T cell activation and maturation status in the different GSK-3 backgrounds. In addition, determination of the pathways downstream of the two isoforms and further mechanistic studies of T cell migration and tumor infiltration will reveal the pathways by which GSK-3 regulates T cell activity. These mechanistic studies were beyond the scope of the current study.

## STAR★Methods

### Key resources table

**Table:** 

REAGENT or RESOURCE	SOURCE	IDENTIFIER
**Antibodies**		

anti-CD8α (clone, 53-6.7)	ebioscience	CAT#12-0081-81; RRID:AB_465529
anti-CD4 (clone, RM4–5)	ebioscience	CAT#MCD0420; RRID:AB_10373704
IFN gamma Monoclonal Antibody (XMG1.2)	ebioscience	CAT#45-7311-82; RRID:AB_1107020
CD3e Monoclonal Antibody (145-2C11)	ebioscience	CAT#17-0031-82; RRID:AB_469315
Granzyme B Monoclonal Antibody (NGZB), PE-Cyanine7	ebioscience	CAT# 25-8898-82; RRID:AB_10853339
Recombinant Anti-CD4 antibody [EPR19514]	Abcam plc	CAT#ab183685; RRID:AB_2686917
Recombinant Anti-CD8 alpha antibody [EPR21769]	Abcam plc	CAT#ab217344; RRID:AB_2890649
PD-1 (D7D5W) XP® Rabbit mAb (Mouse Specific)	Cell Signaling Technology	CAT#84651S; RRID:AB_2800041
FoxP3 (D6O8R) Rabbit mAb	Cell Signaling Technology	CAT#12653S; RRID:AB_2797979

**Critical Commercial Assays**		

RNeasy Mini kit	Qiagen	Cat# 74104
TaqMan Reverse Transcription Reagents	Applied Biosystems	Cat# N808-0234
SYBR® Green PCR Master Mix	Applied Biosystems	Cat# 4309155
ImmPACT® DAB Substrate, Peroxidase (HRP)	Vector	CAT#SK-4105
ImmPRESS® HRP Horse Anti-Mouse IgG PLUS Polymer Kit, Peroxidase	Vector	CAT#MP-7802

**Experimental Models: Cell Lines**		

B16-F10-Luc2 (ATCC® CRL-6475-LUC2™)	ATCC	Cat# CRL-6475-LUC2
EL4 (ATCC® TIB-39™)	ATCC	CAT#TIB-39

**Experimental Models: Organisms/Strains**		

Mouse: 012837 - B6.Cg-Tg(Lck-icre)3779Nik/J	Jackson Laboratories	CAT#012837
Mouse:*Gsk3a*-flox/flox-*b*-flox/flox	James Woodgett	

**Oligonucleotides**		

PD-1-FW, 5-CCGCCTTCTGTAATGGTTTGA-3;	Thermo Fisher Scientific	https://www.thermofisher.com/
PD-1-RV, 5-GGGCAGCTGTATGATCTGGAA-3;	Thermo Fisher Scientific	https://www.thermofisher.com/
GAPDH-FW, 5-CAACAGCAACTCCCACTCTTC-3;	Thermo Fisher Scientific	https://www.thermofisher.com/
GAPDH- RW, 5- GGTCCAGGGTT TCTTACTCCTT-3	Thermo Fisher Scientific	https://www.thermofisher.com/
Tbet-FW, 5-GATCGTCCTGCAGTCTCTCC-3;	Thermo Fisher Scientific	https://www.thermofisher.com/
Tbet-RW, 5-AACTGTGTTCCCGAGGT GTC-3;	Thermo Fisher Scientific	https://www.thermofisher.com/

**Software and Algorithms**		

Prism	GraphPad	https://www.graphpad.comRRID:SCR_002798
CytExpert	Beckman Coulter	https://www.mybeckman.uk/flowcytometry/instruments/cytoflex/software
Aperio and Imagescope software	Leica Biosystems	www.Leicabiosystems.com

### Resource availability

#### Lead contact

Further information and requests for resources and reagents should be directed to and will be fulfilled by the Lead Contact, Dr. Alison.Taylor (a.taylor1@leeds.ac.uk).

#### Materials availability

This study did not generate any new unique reagents.

#### Data and code availability

This study did not generate/analyse any datasets/code.

### Experimental model and subject details

#### Animals

*Gsk3α*-flox/flox-*β*−flox/flox mice were crossed with an Lck-Cre line (Jackson labs) both on a C57BL/6J background. Mice heterozygous for the floxed genes were backcrossed to give mice which were homozygous for the floxed gene and hemizygous for lck-cre. Mice have been backcrossed for more than 10 generations to establish three separate breeding colonies of *Gsk3α*-flox/flox-*β*−flox/flox-Lck-Cre+ (*Gsk3ab* cKO), *Gsk3α*-flox/flox-Lck-Cre+ (*Gsk3a* cKO) and *Gsk3β*-flox/flox-Lck-Cre+ (*Gsk3b* cKO) mice. Breeding mice hemizygous for Lck-cre resulted in litters consisting of approx. 50% floxed-Lck-cre+ animals and 50% floxed-Lck-cre- mice. The latter were used as wild type littermates (containing floxed genes without Lck-cre) which were used as controls alongside Lck-cre only mice. Both female and male animals aged between 6 and 10 weeks were randomly subjected to the experiments and there was no difference in experimental results due to sex differences. The research was regulated under the Animals (Scientific Procedures) Act 1986 Amendment Regulations 2012 Home Office UK PPL Nos. 70/7544 and P0CFA732A.

#### Primary T cell cultures

Spleen cells were treated with a hypotonic buffer with 0.15M NH4CL, 10mM KHCO3 and 0.1mM EDTA, pH 7.2 to eliminate red blood cells before suspension in RPMI 1640 medium supplemented with 10% FCS, 50uM beta-mercaptoethanol, sodium pyruvate, 2 mM L-glutamine, 100 U/ml penicillin and streptomycin (GIBCO). T cells were isolated from tumor-infiltrating cells, spleen and lymph node samples using negative section of magnetically labeled cells (Miltenyi). In some cases, whole lymphocyte samples were used for flow cytometry to determine effect on other cell sub-types.

#### Cell lines

Tumor cells included B16 F10 melanoma and EL4 lymphoma cells (obtained from the ATCC). Cell lines were cultured in DMEM medium supplemented with 10% FCS, 50uM beta-mercaptoethanol, sodium pyruvate, 2 mM L-glutamine, 100 U/ml penicillin and streptomycin (GIBCO). Each cell line was grown to achieve adequate numbers for freezing, followed by repeated thawing for use in the described experiments. The length and time between thawing and use in experiments was on average 2-3 weeks. The cell lines were authenticated by means cell surface staining and flow cytometry for characteristic markers and by their growth properties as described in the literature. Cell cultures were occasionally tested for mycoplasma (last tested in 2011).

### Method details

#### Flow cytometry

Flow cytometry of antibody staining of surface receptors was conducted by suspending 10^6^ cells in 100μl PBS and adding antibody (1:100) for 2hr at 4°C. Cells were then washed twice in PBS and in some cases suspended in 100μl PBS with secondary antibody for a further 1h at 4°C. Cell staining was analyzed on a Beckman Coulter CytoFLEX S flow cytometer and by CytExpert software. For intracellular staining, cells were fixed in 4% paraformaldehyde (PFA), permeabilized with 0.3% saponin (Sigma–Aldrich) and stained with the desired antibody in saponin containing PBS for 2hr at 4°C, followed by a secondary Ab incubation where primary antibodies were not conjugated.

#### Antibodies and reagents

The following antibodies were used in experiments; conjugated antibodies for flow cytometry: anti-CD8α (clone, 53-6.7), anti-CD4 (clone, RM4–5), anti-IFNγ, anti-CD3, anti-Granzyme B (ebioscience). Antibodies for IHC: anti-PD1 and anti-FOXP3 (Cell Signaling Technology), anti-CD8 and anti-CD4 (Abcam)

#### Quantitative real-time polymerase chain reaction (PCR)

Single-strand cDNA was synthesized with an RT-PCR kit (Qiagen) according to the manufacturer’s instructions. Reverse transcription was performed using the RNA polymerase chain reaction (PCR) core kit (Applied Biosystems). Quantitative real-time PCR used SYBR green technology (Applied biosystems) on cDNA generated from the reverse transcription of purified RNA. After preamplification (95°C for 2 min), the PCRs were amplified for 40 cycles (95°C for 15s and 60°C for 60s) in a sequence detection system (PE Prism 7000; Perkin-Elmer Applied Biosystems). The exponential phase, linear phase and plateau phase of PCR amplification were carefully monitored to ensure a measurement of real time transcription (33). mRNA expression was normalized against GAPDH expression using the standard curve method.

PD-1-FW, 5-CCGCCTTCTGTAATGGTTTGA-3;

PD-1-RV, 5-GGGCAGCTGTATGATCTGGAA-3;

Tbet-FW, 5-GATCGTCCTGCAGTCTCTCC-3;

Tbet-RW, 5-AACTGTGTTCCCGAGGT GTC-3;

GAPDH-FW, 5-CAACAGCAACTCCCACTCTTC-3;

GAPDH- RW, 5- GGTCCAGGGTT TCTTACTCCTT-3

#### Immunohistochemistry

IHC was performed on formalin-fixed paraffin-embedded tissue sections. Following dewaxing, rehydration, and endogenous peroxidase blocking by a 3% solution of H2O2 (Sigma) for 20 min. Antigen retrieval was performed using citrate or Tris-EDTA buffers as per antibody datasheets. Nonspecific antibody binding was blocked by incubation with either horse or goat serum (Vector). Primary antibodies were diluted 1:500 (PD1, FOXP3) or 1:200 (CD4, CD8) in Antibody dilution buffer (Life Technologies #003218) and were applied for 1h at room temp. Following two washes in TBS, ready-to-use secondary antibodies were applied (ImmPRESSTM HRP reagent kits, Vector). Sections were washed twice in TBS and ImmPACTTM DAB (Vector) was used to detect immunolabeling. Sections were counterstained in hematoxylin, and following dehydration and clearing in xylene, were mounted in distyrene/plasticizer/xylene. Sections were scanned using Aperio and Imagescope software (Leica Biosystems) was used to determine percentage positive cells.

#### Melanoma lung tumor establishment

B16 melanoma cells (2 x 10^5^ taken from the log phase of *in vitro* growth) OVA-peptide pulsed or non-pulsed) were transferred intravenously into GSK-3 cKO, wt littermates or lck-cre mice 8-10 weeks old.

Live imaging was performed at the timepoints indicated. Mice were injected intraperitoneally with luciferin (2 ug per mouse), anaesthetized with isoflurane and scanned with an IVIS Lumina (Caliper Life Sciences). For quantitative comparisons, we used Living Image software (Caliper Life Sciences) to obtain the maximum radiance (photons per s per cm2 per steradian, i.e. photons s−1 cm−2 sr−1) over each region of interest, relative to a negative control region.

#### Intradermal tumor establishment

EL4 or B16 tumor cells were taken from the log phase of *in vitro* growth (approx. 70% confluency). They were then washed and injected into mice (typically 3 x 10^6^ cells for EL4 and 2 x 10^5^ for B16 cells). Tumors were clearly visible after 1 week and grew progressively in an encapsulated fashion. Induced tumors were measured on a daily basis using a vernier caliper. Tumors, spleens and lymph nodes were harvested as indicated, or when the tumor reached a maximum diameter of 15mm.

#### Isolation of tumor-infiltrating lymphocytes (TILs)

Solid tumors or nodules from lungs were harvested from mice at the time indicated. Tissue was disrupted using a blade and then incubated in HBSS solution containing 200units/ml of collagenase at 37^o^C for 2 hrs. Tissue was then passed through a strainer and cells collected and layered onto ficoll before centrifugation. Tumor-infiltrating cells were then collected from the lymphocyte layer.

### Quantification and statistical analysis

The mean and SE of each treatment group were calculated for all experiments. The number of samples is indicated in the figure legends. Unpaired Student’s t tests or ANOVA tests were performed using the InStat 3.0 software (GraphPad). In certain instances, statistics were done using 2-way ANOVA, or by non-parametric Mann Whitney at each time point. ∗ P < 0.05, ∗∗ P < 0.01, ∗∗∗ P < 0.001. Log rank tests for Kaplan-Meier survival data were performed to determine significant differences.
