# Supplementary material for: Non-redundant activity of GSK-3α and GSK-3β in T cell-mediated tumor rejection
Source: iScience. 2021 May 19;24(6):102555. doi: 10.1016/j.isci.2021.102555 (PMC8188550; doi:10.1016/j.isci.2021.102555)
Supplement: Document S1. Figure S1 [file mmc1.pdf]

iScience, Volume *24*

Supplemental information

Non-redundant activity of GSK-3α and GSK-3β

in T cell-mediated tumor rejection

Lynette Steele, Aarren J. Mannion, Gary Shaw, Kenneth A. Maclennan, Graham P. Cook, Christopher E. Rudd, and Alison Taylor

Figure S1. Tumour rejection in *Gsk-3b* cKO is associated with higher numbers of CD4+ T cells and increased FoxP3 expression, related to Figure 6.

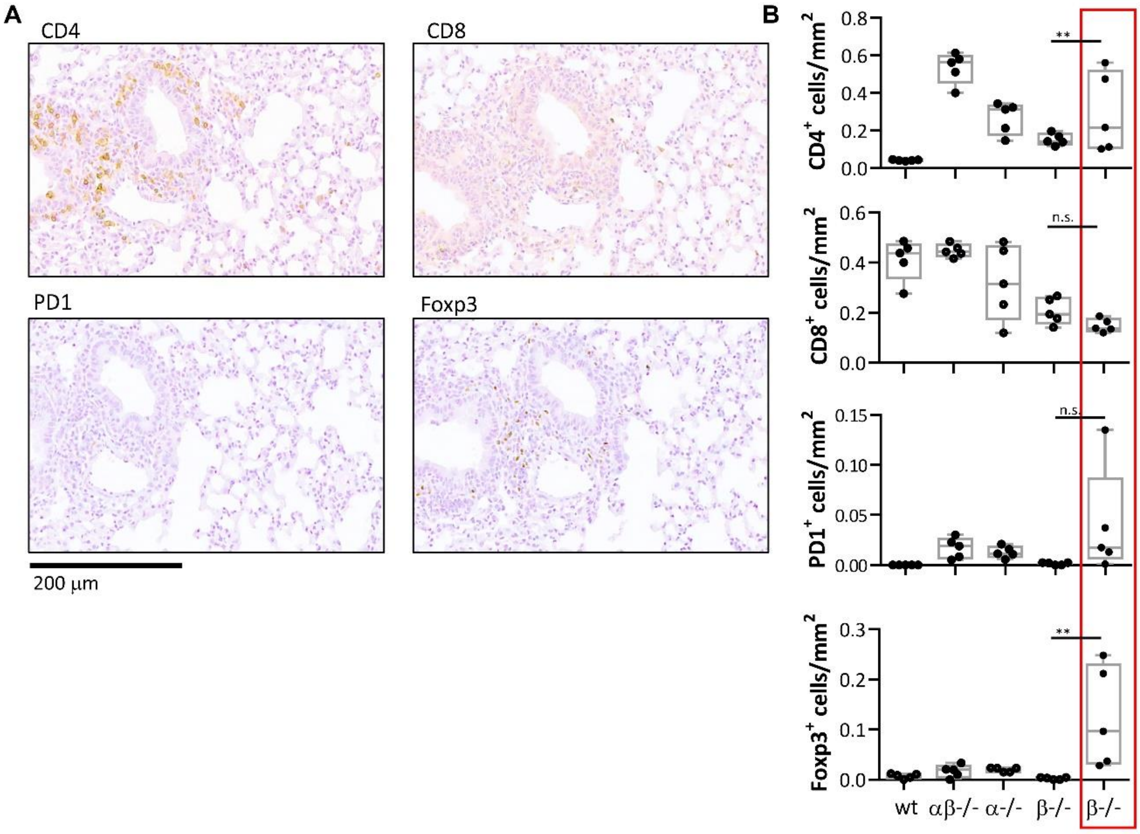

Immunohistochemistry of lung tissue sections from a *Gsk3b* cKO mouse following B16 tumour cell rejection. (**A**) Sections demonstrating CD4, CD8, PD1 and FOXP3 expression. (**B**) The number of positive cells were quantified from 5 different areas using ImageScope Software
